# LAMP3 expression in the liver is involved in T cell activation and adaptive immune regulation in hepatitis B virus infection

**DOI:** 10.3389/fimmu.2023.1127572

**Published:** 2023-03-16

**Authors:** Zilong Wang, Xiaoxiao Wang, Rui Jin, Feng Liu, Huiying Rao, Lai Wei, Hongsong Chen, Bo Feng

**Affiliations:** ^1^ Peking University People’s Hospital, Peking University Hepatology Institute, Beijing Key Laboratory of Hepatitis C and Immunotherapy for Liver Diseases, Beijing International Cooperation Base for Science and Technology on NAFLD Diagnosis, Beijing, China; ^2^ Beijing Tsinghua Changgung Hospital, Tsinghua University, Beijing, China

**Keywords:** LAMP3 1, T cell 2, immune regulation 3, liver 4, hepatitis B virus

## Abstract

**Background:**

The disease burden caused by chronic hepatitis B virus (HBV) infection is still heavy, and the current treatment scheme has not achieved a complete cure. Changes in natural and adaptive immunity usually accompany chronic HBV infection. As a marker expressed on dendritic cells (DCs), whether lysosome-associated membrane glycoprotein 3 (LAMP3) participates in chronic HBV infection deserves further analysis.

**Methods:**

We retrieved chronic HBV infection transcriptional information from the Gene Expression Omnibus (GEO) database. The LAMP3 expression in the liver of patients with chronic hepatitis B (CHB) was analyzed in three GEO datasets and confirmed in our validation cohort (27 patients with CHB). Differentially expressed genes were obtained from one CHB cohort by comparing LAMP3^high^ and LAMP3^low^ expression subgroups. These genes underwent Gene Ontology, Kyoto Encyclopedia of Genes and Genomes analysis, and Gene Set Enrichment Analysis to decipher the influence of LAMP3 on the biological process and immunity changes in HBV infection. Furthermore, we investigated the potential relationship between LAMP3 levels, the abundance of infiltrating immune cells, and liver dysfunction.

**Results:**

Compared to healthy controls, LAMP3 expression was upregulated in the transcriptional profiles of the liver in patients with CHB. The high LAMP3 expression was related to T cell activation and the chemokine signaling pathway. The LAMP3 gene was positively linked to marker sets of infiltrating activated regulatory T cells (Treg), T cell exhaustion, monocytes, and DCs. Moreover, CHB patients with high LAMP3 expression had unfavorable liver dysfunction.

**Conclusions:**

LAMP3 is a gene related to HBV infection, which might be involved in HBV infection by regulating T cell activation and adaptive immune response.

## 1 Introduction

Chronic hepatitis B (CHB) infection is a globally recognized public health problem, estimated to affect approximately 300 million people worldwide (1–4). Despite the successful introduction and practical application of an HBV vaccine, the disease burden of HBV-related liver disease is still heavy (5). CHB is directly related to the occurrence and development of cirrhosis, liver cancer, and the long-term morbidity associated with liver disease. Additionally, as the second most lethal cancer in the world, liver cancer accounts for 600,000 deaths annually (5, 6).

Previous basic and clinical studies have analyzed chronic HBV infection’s characteristics, natural history, and immunological pathogenesis (4, 6, 7). Currently, the primary therapeutic strategy against chronic HBV infection is suppressing viral replication, reducing liver injury caused by the virus, and improving patients’ life quality (8). The treatment of CHB infection mainly focuses on nucleoside (nucleotide) analogs that inhibit HBV DNA replication, reverse transcription, and pegylated interferon that activates the adaptive immune response. These treatment schemes can achieve a partial or functional cure for chronic HBV infection. However, these schemes are still unable to achieve a complete or sterilizing cure (9). Recently, researchers presented various ideas on hepatitis B immunotherapy, including the TLR 7/8 agonist and retinoic acid-inducible gene I, immune checkpoint inhibitors, therapeutic vaccines, and peginterferon lambda (10–15). However, these are all trial treatments without adequate clinical evidence.

Recent studies have provided a new perspective on immunological markers as therapeutic agents for chronic liver diseases. Lysosome-associated membrane glycoprotein 3 (LAMP3) is a glycosylated membrane protein located on the 3q chromosome (16, 17). As a member of the lysosomal-associated membrane protein family, LAMP3 is usually expressed in lymphoid organs (18). At the cellular level, LAMP3 is a marker for mature dendritic cells (DCs) in humans, and it is upregulated upon DC activation and maturation (19). However, it is unclear whether LAMP3 is involved in chronic HBV infection. In this study, we analyzed the Gene Expression Omnibus (GEO) databases and our cohort, explored the correlation between LAMP3 and HBV infection, and investigated related immunological changes.

## 2 Materials and methods

### 2.1 Data resources

We collected three independent transcriptional profiles from the GEO database, including the liver samples of healthy control and patients with CHB. Furthermore, as a representative of non-viral hepatitis, one RNA-seq dataset of patients with nonalcoholic steatohepatitis (NASH) was also used for validation in other hepatitis. **Table 1** showed detailed information on these four cohorts. The LAMP3 expression between patients with CHB and healthy controls was analyzed through the “edgeR” package.

**Table 1:** Details of four GEO cohorts used in this study.

GEO series	Contributors	Year	Control (N)	CHB/NASH (N)	Platform
GSE83148	Zhou W, Ma Y, Wang J, Zheng H, Liu J	2017	6	122	Affymetrix Human Genome U133 Plus 2.0 Array
GSE159413	Nishio A, Rehermann B	2020	19	12	NanoString nCounter GX Human Immunology v2
GSE166759	Yao J, Diao H	2021	3	3	Agilent-079487 Arraystar Human LncRNA microarray V4
GSE89632	Allard JP, Arendt BM, Comelli EM, Ma DW, Lou W, Teterina A, Kim T, Fung SK, Wong DK, McGilvray I, Fischer SE	2016	19	24	Illumina HumanHT-12 WG-DASL V4.0 R2 expression beadchip

### 2.2 Differentially expressed genes analysis

According to the median counts of LAMP3 expression, patients with CHB were divided into LAMP3^high^ and LAMP3^low^ expression groups. DEGs (Log2 [Fold change] >1, adjusted p value<0.05) between these two groups were extracted and analyzed using R 4.1.2 (R Foundation for Statistical Computing, Vienna, Austria) for Gene Ontology (GO) function, Kyoto Encyclopedia of Genes and Genomes (KEGG) pathway, and Gene Set Enrichment Analysis (GSEA).

### 2.3 Gene correlation analysis

Pearson correlation analysis were applied to evaluate the genes related to LAMP3 expression. The associations of LAMP3 expression with the adaptive immune response-related genes were analyzed by the “cor.test” function of R studio. The “ggcatterstats” function in the “ggstatplot” package generated the dot plot. Finally, the analyzed results were visualized in Glue modules by Cytoscape 3.9.1.

### 2.4 Validation cohort

We collected liver tissues of 27 patients with CHB and 5 healthy controls (normal liver tissue of patients with hepatic hemangioma) to validate the bioinformatic results. The detailed information of all participants showed in **Table 2**. All paraffin-embedded formalin-fixed liver biopsy samples were obtained from the Peking University People’s Hospital Hepatology Department. This study was approved by the Ethical Committees of Peking University People’s Hospital (no. 2020PHE081), and all participants signed the written informed consent forms.

**Table 2:** Clinical information of 27 patients with CHB.

	Control (n=5)	All CHB (n=27*)	CHB (IT, n=3)	CHB (IA, n=5)	CHB (LR, n=11)	CHB (RA, n=3)
Sex (M/F)	3/2	22/5	3/0	5/0	9/11	1/2
Age (y)	49	43	47	40	45	41
ALT (U/L)	35	94	49	294	30	192
AST (U/L)	30	99	40	334	26	185
ALP (U/L)	56	105	85	111	87	112
GGT (U/L)	38	78	20	177	33	68
TBIL	15.3	31.2	13.4	134.6	16.5	19.1
HBsAg	–	+	+	+	+	+
HBsAb	–	–	–	–	–	–
HBeAg	–	+/–	+	+	–	–
HBeAb	–	+/–	–	–	+	+
HBcAb	–	+	+	+	+	+
HBV DNA (IU/ml)	–	6.29 10E+06	3.94 10E+07	3.91 10E+06	4.73 10E+02	2.84 10E+04
LAMP3+ cells (n, ×200)	1	7	4	10	5	17

Ps: Sex showed the number of males/females of CHB patients, ALT, AST, ALP, GGT, HBV DNA and the LAMP3+ cells showed the mean of each item. IT, Immune tolerant phase; IA, Immune activation; LR, Low replication; RA, Reactivation; ALT, alanine aminotransferase; AST, aspartate aminotransferase; ALP, alkaline phosphatase; GGT, γ-glutamyl transpeptidase; y, year; CHB, chronic hepatitis B. *There were some missing data from certain samples.

### 2.5 Staining and histological evaluation

Subsequently, immunohistochemistry (IHC) was performed using primary antibody anti-LAPM3 (1:200, cat# ab271053, Abcam) to validate the expression in our cohort. Five fields (×200) were randomly selected and captured for analysis in each sample. The number of cells in the portal tract (per portal tract area) was quantified.

### 2.6 Statistical analysis

All statistical analysis was completed by SPSS 20.0 (Chicago, IL, USA) and R 4.1.2 (Vienna, Austria). The differences of transcriptional levels between control and CHB patients, or LAMP3^high^ and LAMP3^low^ expression groups were analyzed by the Mann–Whitney U test. Two paired p < 0.05 was assumed significant statistically.

## 3 Results

### 3.1 LAMP3 expression upregulated in transcriptional profiles of patients with CHB

**Figure 1:**
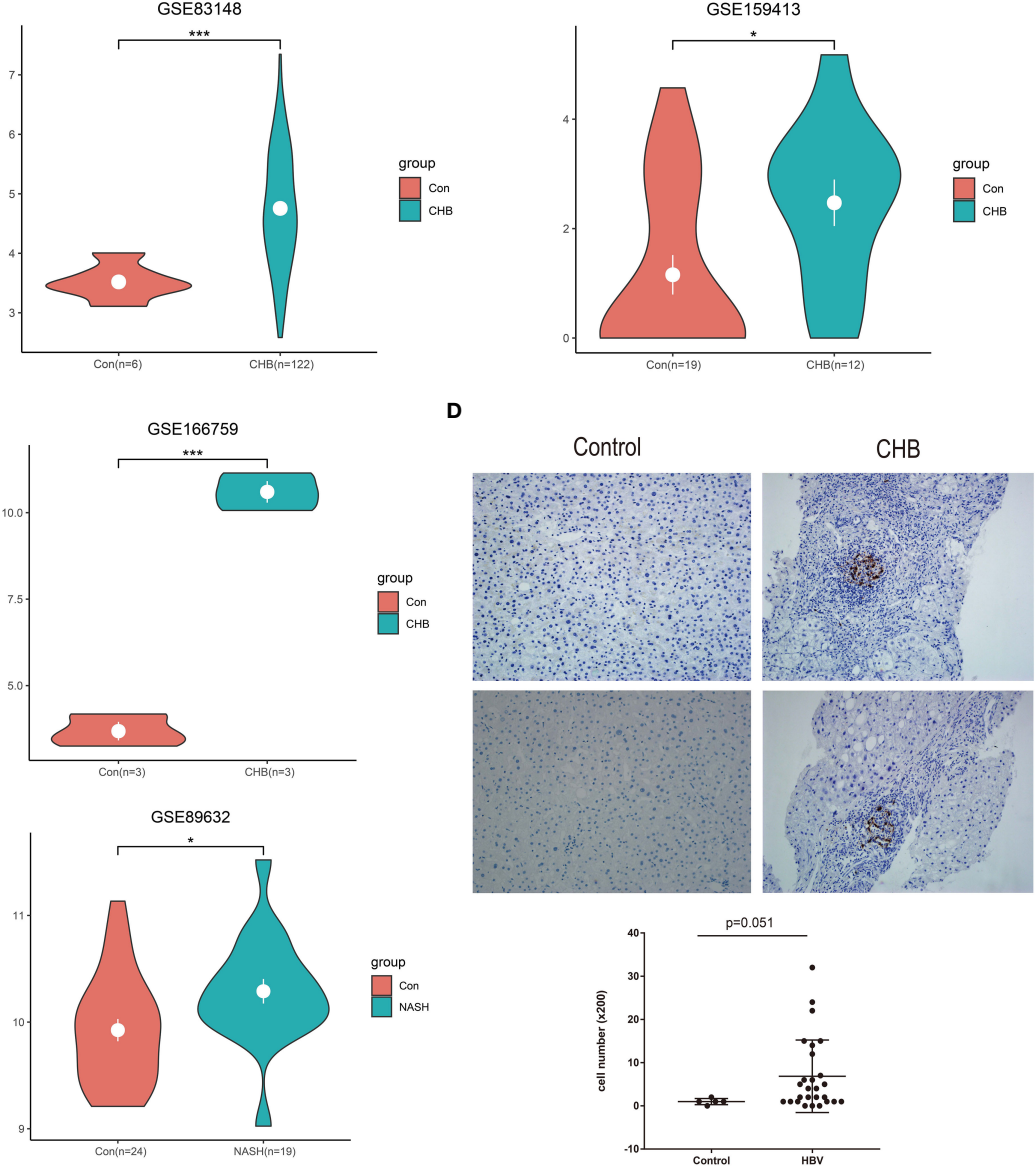
Upregulated LAMP3 expression in patients with CHB. **(A–C)** The comparison of LAMP3 transcriptional levels between patients with CHB and healthy controls in three GEO databases. **(D)** The expression of LAMP3 in our validation cohort. **(E)** LAMP3 expressions in patients with NASH and controls. *p <0.05, **p <0.01, ***p <0.001. con, Control; CHB, chronic hepatitis B; GEO, Gene Expression Omnibus; NASH, nonalcoholic steatohepatitis; LAMP3, lysosome-associated membrane glycoprotein 3.

In total, three GEO RNA-seq profiles (GSE83148, GSE159413, and GSE166759) were used to compare the transcriptional expression of LAMP3 between patients with CHB and healthy controls. **Table 1** presented detailed information on these GEO datasets. Analyzed results showed that the mRNA levels of LAMP3 in the livers of patients with CHB increased significantly compared to that in healthy controls (all p<0.05, **Figures 1A–C**). Furthermore, using our cohort, we validated the upregulated LAMP3 protein level in our CHB liver tissues, the representative images showed in **Figure 1D**. We also explored mRNA levels in a transcriptional profile of patients with NASH, representing a sterile inflammatory and immune-activated state. Similarly, the levels of LAMP3 mRNA in the liver of patients with NASH was significantly higher than that of healthy controls (**Figure 1E**).

### 3.2 LAMP3 upregulation was associated with T cell activation and adaptive immune regulation in patients with CHB

**Figure 2:**
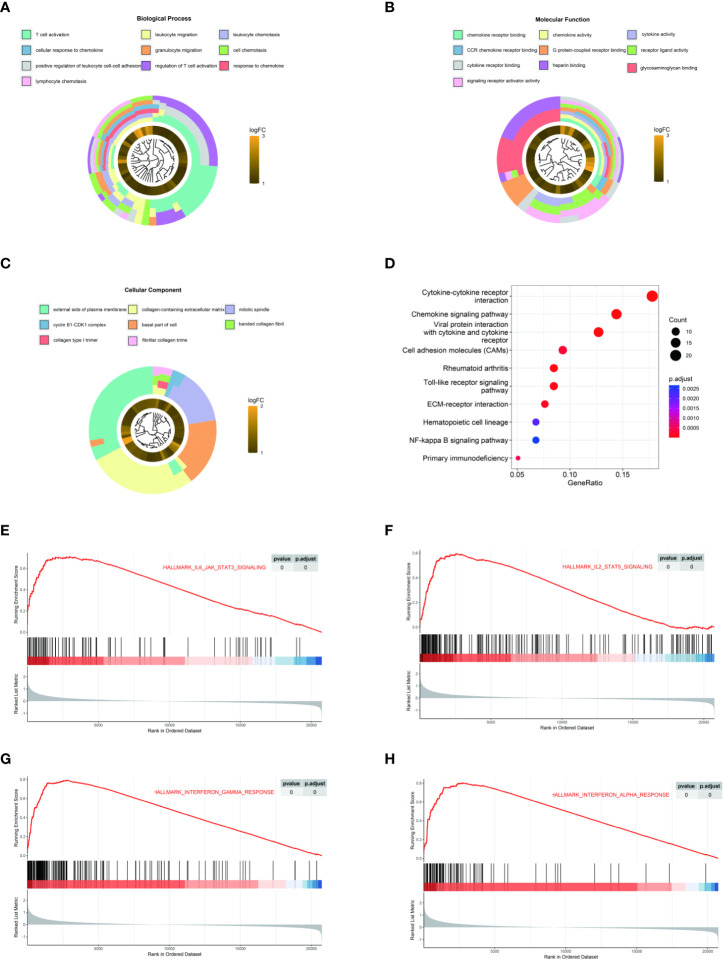
LAMP3 upregulation is associated with T cell activation and adaptive immune regulation. **(A–D)** GO analysis and KEGG pathway enrichment of 216 upregulated DEGs. **(E–H)** GSEA showed that LAMP3 is positively associated with various bioprocesses of T cells. DEGs, differentially expressed genes; KEGG, Kyoto encyclopedia of genes and genomes; GSEA, Gene Set Enrichment Analysis; LAMP3, lysosome-associated membrane glycoprotein 3.

To explore the potential function of LAMP3 in CHB infection, we equally divided patients from one CHB cohort (GSE83148) into two groups (LAMP3^high^ and LAMP3^low^) according to the median expression levels of LAMP3. DEGs between LAMP3^high^ and LAMP3^low^ subgroups were then analyzed. Biological processes and molecular functions in GO enrichment showed that the upregulated DEGs were mainly associated with T cell activation, cell response to chemokine, cytokine, and chemokine receptor binding (**Figures 2A–C**). KEGG enrichment analysis indicated that the upregulated genes were primarily distributed in the cytokine-cytokine receptor interaction and chemokine signaling pathway (**Figure 2D**). GSEA analysis explored the signaling pathways associated with upregulated LAMP3 mRNA levels. The analyzed results showed that genes in the LAMP3^high^ cohort were mainly enriched in the cytokine signaling pathway. For example, IL2-STAT5, IL6-JAK-STAT signaling, and interferon α and γ response were enriched, similar to that observed in GO and KEGG analyses (**Figures 2E-H**).

**Figure 3:**
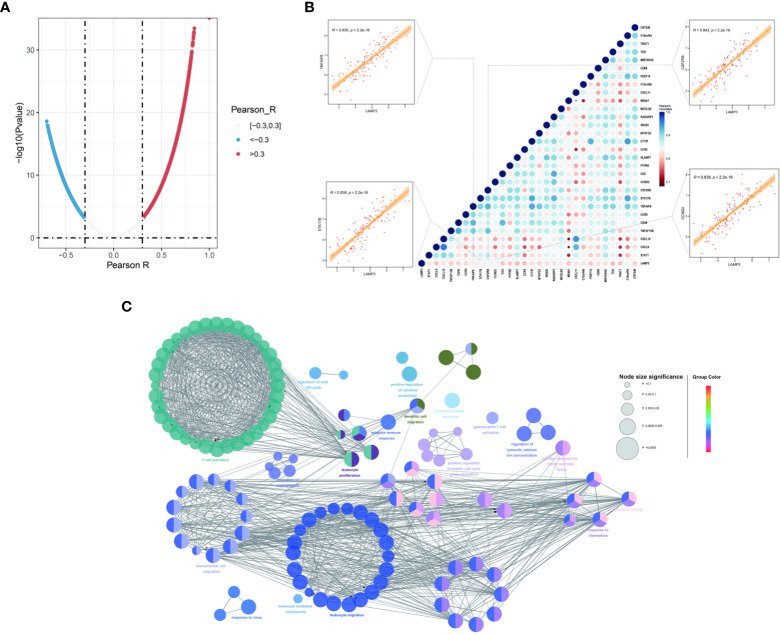
Associations between LAMP3 and key genes related to immune cell regulation. **(A)** The spoon-shaped plot shows genes related to LAMP3 expression calculated by Pearson correlation analysis. **(B)** Correlation between immunocyte regulatory genes and LAMP3, and their impact on HBV infection. **(C)** Visualization of the interaction network of genes strongly associated with LMAP3 by Cytoscape (ClueGO module). Node size, p value; Node color, gene groups. HBV, Hepatitis B virus; LAMP3, lysosome-associated membrane glycoprotein 3.

Co-expression analysis was performed to characterize the genes associated with the expression of LAMP3 in the CHB cohort (**Figure 3A**). These genes were strongly associated with LAMP3 expression and analyzed further (r>0.8, p<0.05, **Figure 3B**). We found that the genes related to T cell activation, DCs, macrophage maturation, and cytokine release were significantly related to the expression of LAMP3. The CCND2, CSF2RB, STK17B, and TNFAIP8 genes were primarily expressed on T- and DC cells (all r>0.83, p<0.05) (**Figure 3B**). Additionally, using GlueCo, we could visualize the interactive network of genes that were in the same biological processes and functionally related. The significantly related biological functions were leukocyte proliferation, migration, and T cell activation (p<0.001, **Figure 3C**).

### 3.3 Relationship between LAMP3 expression and marker genes sets of various immune cells

**Figure 4:**
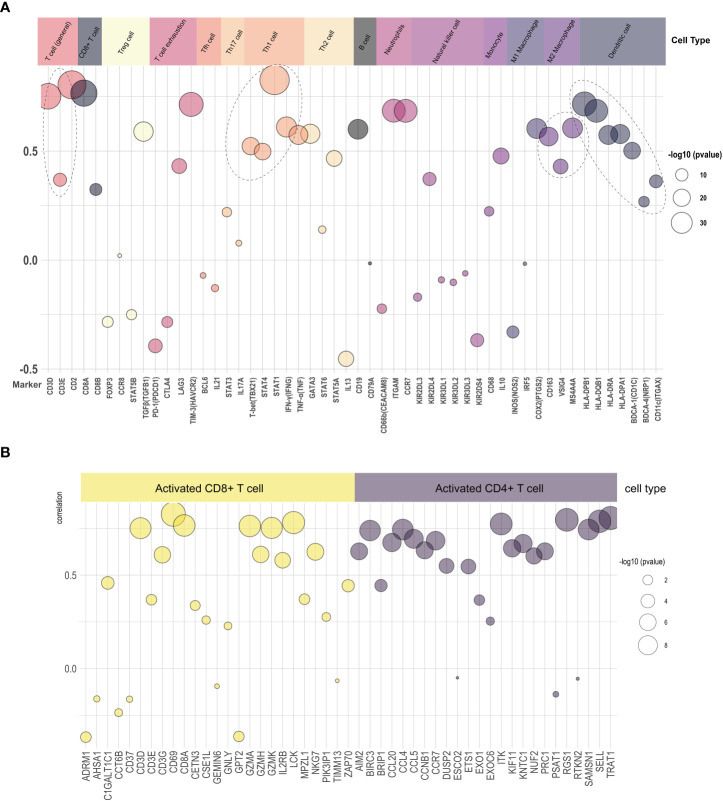
Relationship of LAMP3 expression with marker gene sets of various immune cells. **(A)** The association among LAMP3 level with marker genes in immune cells and **(B)** CD4+ T cells and CD8+ T cells. LAMP3, lysosome-associated membrane glycoprotein 3; Circle size indicated -log10 (p value), Y-axis displayed the correlation coefficient.

We explored the relationship of LAMP3 mRNA levels to the abundance of infiltrating immune cells, including T cells, B cells, neutrophils, natural killer cells, monocytes, macrophages, and DCs (**Figure 4A**). Remarkably, there was a close relationship among markers of Treg cells (TGFβ), T cell exhaustion (TIM3), and Th1 cells (STAT1, IFN-γ). Moreover, the mRNA levels were positively associated with general T cell markers and CD8+ T cells. In addition, the DC markers (CD1C, HLA-DPB1), macrophages, monocytes (CD86, CSF1R), and neutrophils (ITGAX, CCR7) showed a close correlation with LAMP3 levels (**Figure 4A**). After T cell was divided into CD4+ T cells and CD8+ T cells, the mRNA levels of RGS1 and TRAT1 in CD4^+^T cells and CD69 and LCK in CD8^+^T cells were correlated with LAMP3 level significantly (**Figure 4B**).

### 3.4 LAMP3 expression was associated with liver dysfunction in HBV infection

**Figure 5:**
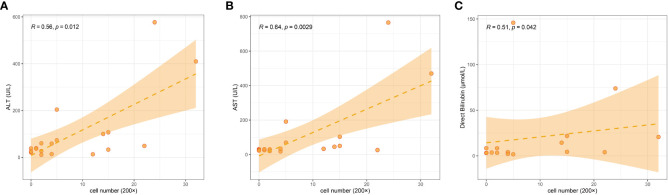
Correlation among numbers of LAMP3+ cells in liver section with serum markers of liver dysfunction in patients with CHB. **(A)** ALT, **(B)** AST, **(C)** DBIL. ALT, alanine aminotransferase; AST, aspartate aminotransferase; DBIL, direct bilirubin; CHB, chronic hepatitis B.

We counted LAMP3+ cells in each portal area under a 200× visual field and analyzed the correlation between LAMP3+ cells and serum markers of liver function injury. The results indicated that the number of LAMP3+ cells was positively correlated with the levels of alanine aminotransferase (ALT), aspartate aminotransferase (AST), and direct bilirubin (DBIL) (**Figures 5A–C**). In addition, there were differences in the expression level of LAMP3 in patients at different natural history stages of HBV infection. The expression level of LAMP3 in patients in immune activation (IA) and reactivation (RA) stage is relatively high (**Table 2**; **Supplementary Figure 2**).

## 4 Discussion

Chronic HBV infection and persistence are usually accompanied by changes in natural and adaptive immunity, particularly in T- and DC cells. In this study, by analyzing GEO datasets, we found that the LAMP3 gene was associated with chronic HBV infection, and the upregulation of LAMP3 expression was mainly involved in T cell activation and adaptive immune response. Additionally, the increased LAMP3 expression was positively associated with liver dysfunction caused by HBV infection.

In our research, LAMP3 mRNA and protein levels increased in the liver of patients with CHB compared to healthy controls. Previous studies showed that LAMP3 was involved in viral infection and the formation of various biological processes. For instance, in nonalcoholic fatty liver disease (NAFLD), the overexpression of LAMP3 substantially increased the lipid accumulation of hepatocytes (20), in our study, we also found that the expression of LAMP3 in the liver of NASH patients was significantly higher than that in healthy controls. From the perspective of virus infection, LAMP3 also participated in the replication of the influenza virus. Influenza viral infection could induce the upregulation of LAMP3 expression (21).

Furthermore, in other viral infections, e.g., hepatitis C virus and human papillomavirus, LAMP3 is also specifically induced as a classical interferon-stimulated gene (22, 23). Moreover, LAMP3 is overexpressed in various tumors and is related to a poor prognosis and tumor metastasis (24–26). Therefore, the expression of LAMP3 is involved in various viral infections and tumor prognoses.

We also analyzed the biological mechanism of LAMP3 participation in HBV infection. The GEO database was analyzed, and we found that the upregulated LAMP3 expression in HBV-infected patients mainly participated in T cell activation and adaptive immune response. Previous studies showed that LAMP3 was primarily expressed in DC cells, and LAMP3 participated in major histocompatibility complex class II-restricted antigen presentation and the processing of exogenous antigens (27). LAMP3 is involved in HBV infection, possibly because LAMP3 on DC cells promoted T cell activation and adaptive immunity changes. Zhang et al. and Oh et al. proposed that LAMP3+ DCs expressed many ligands to interact with receptors on T cells, possibly being the most active immune regulators of lymphocytes (28, 29).

Currently, research on the interaction between LAMP3+ DCs and T cells is mainly focused on the tumor field, which might provide a basis for the understanding that LAMP3 participates in T cell activation and immune regulation during HBV infection. For example, in hepatocellular carcinoma, LAMP3+ DCs were positively correlated with the infiltration of exhausted CD8+ T cells and Tregs (30). In gastric cancer, LAMP3+ DCs were predicted to deliver attracting and activating signals to lymphocytes. However, LAMP3+ DCs inhibited anti-tumor T cell activity through a high expression of PD-L1 (31). In lymph node metastasized tumors, LAMP3+ DCs showed a stronger interaction with Tregs to enhance immunosuppression (32). In pancreatic adenocarcinoma, urothelial bladder carcinoma, and cutaneous T-cell lymphoma, LAMP3+ DCs also promote immune tolerance and immunosuppression through interacting with CD8+T or tumor-infiltrating Tregs (33–35). LAMP3+ DCs highly expressed CD80 and CD86, through interaction of CD80-CD28, CD80-CTLA4, CD86-CD28, and CD86-CTLA4, which might modulate CXCL13+/CD4+, and FOXP3+/CD4+ Tregs activities (36, 37). This indicated a close relationship among markers of Treg cells, T cell exhaustion, and Th1 cells, which illustrated the tight association between LAMP3 levels and the exhausted status of T cells. These results suggested that LAMP3+ DCs triggered T cell activation and exhaustion signaling simultaneously. Although there was no direct evidence, this might be the molecular mechanism of LAMP3 involvement in liver immunity changes and liver function during HBV infection.

## 5 Conclusion

Although no previous studies have reported on the association of LAMP3 with HBV infection, our study adds knowledge to this research gap. Our results revealed that the increased LAMP3 expression could enhance T cell activation and adaptive immune regulation. However, this study had several limitations. First, our explanation of the role of LAMP3 in HBV infection was based on pre-existing data from the GEO database and verified by our cohort; however, we did not confirm the function of LAMP3 in HBV infection by conducting *in vivo* and *in vitro* experiments. Second, the validation sample in this study was a cross-sectional cohort. In the future, we need to explore the application value of LAMP3 in HBV treatment.

## Data availability statement

The original contributions presented in the study are included in the article/**Supplementary Material**. Further inquiries can be directed to the corresponding author.

## Ethics statement

This study was approved by the Ethical Committees of Peking University People’s Hospital (no. 2021PHB122). The patients/participants provided their written informed consent to participate in this study.

## Author contributions

ZW, XW and BF contributed to conception and design of the study. RJ, FL and HR performed the experiments. LW, HC, and BF analyzed and interpreted of data. ZW and XW wrote the first draft of the manuscript. BF made critical revision of the manuscript for important intellectual content. All authors contributed to manuscript revision, read, and approved the submitted version.

## References

[1] Organization. WH. Global hepatitis report. Geneva, Switzerland: World Health Organization (2017).

[2] Fung S Choi HSJ Gehring A Janssen HLA. Getting to hbv cure: The promising paths forward. Hepatology (2022) 76(1):233–50. doi: 10.1002/hep.32314 34990029

[3] Yuen MF Chen DS Dusheiko GM Janssen HLA Lau DTY Locarnini SA. Hepatitis b virus infection. Nat Rev Dis Primers (2018) 4:18035. doi: 10.1038/nrdp.2018.35 29877316

[4] Polaris Observatory C. Global prevalence, treatment, and prevention of hepatitis b virus infection in 2016: A modelling study. Lancet Gastroenterol Hepatol (2018) 3(6):383–403. doi: 10.1016/S2468-1253(18)30056-6 29599078

[5] Asrani SK Devarbhavi H Eaton J Kamath PS. Burden of liver diseases in the world. J Hepatol (2019) 70(1):151–71. doi: 10.1016/j.jhep.2018.09.014 30266282

[6] Liaw YF Chu CM. Hepatitis b virus infection. Lancet (2009) 373(9663):582–92. doi: 10.1016/S0140-6736(09)60207-5 19217993

[7] Maini MK Burton AR. Restoring, releasing or replacing adaptive immunity in chronic hepatitis b. Nat Rev Gastroenterol Hepatol (2019) 16(11):662–75. doi: 10.1038/s41575-019-0196-9 31548710

[8] Lai CL Yuen MF. Prevention of hepatitis b virus-related hepatocellular carcinoma with antiviral therapy. Hepatology (2013) 57(1):399–408. doi: 10.1002/hep.25937 22806323

[9] Lok AS Zoulim F Dusheiko G Ghany MG. Hepatitis b cure: From discovery to regulatory approval. Hepatology (2017) 66(4):1296–313. doi: 10.1002/hep.29323 PMC6294322 28762522

[10] Chan YK Gack MU. Viral evasion of intracellular DNA and rna sensing. Nat Rev Microbiol (2016) 14(6):360–73. doi: 10.1038/nrmicro.2016.45 PMC5072394 27174148

[11] Luk A Jiang Q Glavini K Triyatni M Zhao N Racek T. A single and multiple ascending dose study of toll-like receptor 7 agonist (Ro7020531) in Chinese healthy volunteers. Clin Transl Sci (2020) 13(5):985–93. doi: 10.1111/cts.12791 PMC7485962 32268000

[12] Gane EJ Kim HJ Visvanathan K Kim YJ Nguyen AH Wallin JJ. Safety, pharmacokinetics, and pharmacodynamics of the oral Tlr8 agonist selgantolimod in chronic hepatitis b. Hepatology (2021) 74(4):1737–49. doi: 10.1002/hep.31795 33704806

[13] Ma H Lim TH Leerapun A Weltman M Jia J Lim YS. Therapeutic vaccine brii-179 restores hbv-specific immune responses in patients with chronic hbv in a phase Ib/Iia study. JHEP Rep (2021) 3(6):100361. doi: 10.1016/j.jhepr.2021.100361 34661089 PMC8502773

[14] Riva A Chokshi S. Immune checkpoint receptors: Homeostatic regulators of immunity. Hepatol Int (2018) 12(3):223–36. doi: 10.1007/s12072-018-9867-9 PMC5999155 29740793

[15] Chan HLY Ahn SH Chang TT Peng CY Wong D Coffin CS. Peginterferon lambda for the treatment of hbeag-positive chronic hepatitis b: A randomized phase 2b study (Lira-b). J Hepatol (2016) 64(5):1011–9. doi: 10.1016/j.jhep.2015.12.018 26739688

[16] Gui Y Liu WB Chen H Ma JL Li JS. Expression of Lamp3 and its correlation with clinicopathologic characteristics and prognosis in hepatocellular carcinoma. Int J Clin Exp Pathol (2018) 11(1):367–74. PMC6957939 31938120

[17] Ozaki K Nagata M Suzuki M Fujiwara T Ueda K Miyoshi Y. Isolation and characterization of a novel human lung-specific gene homologous to lysosomal membrane glycoproteins 1 and 2: Significantly increased expression in cancers of various tissues. Cancer Res (1998) 58(16):3499–503. 9721848

[18] Johansson P Corripio-Miyar Y Wang T Collet B Secombes CJ Zou J. Characterisation and expression analysis of the rainbow trout (Oncorhynchus mykiss) homologue of the human dendritic cell marker Cd208/Lysosomal associated membrane protein 3. Dev Comp Immunol (2012) 37(3-4):402–13. doi: 10.1016/j.dci.2012.02.012 22402276

[19] Salaun B de Saint-Vis B Pacheco N Pacheco Y Riesler A Isaac S. Cd208/Dendritic cell-lysosomal associated membrane protein is a marker of normal and transformed type ii pneumocytes. Am J Pathol (2004) 164(3):861–71. doi: 10.1016/S0002-9440(10)63174-4 PMC1613301 14982840

[20] Liao X Song L Zhang L Wang H Tong Q Xu J. Lamp3 regulates hepatic lipid metabolism through activating Pi3k/Akt pathway. Mol Cell Endocrinol (2018) 470:160–7. doi: 10.1016/j.mce.2017.10.010 29056532

[21] Zhou Z Xue Q Wan Y Yang Y Wang J Hung T. Lysosome-associated membrane glycoprotein 3 is involved in influenza a virus replication in human lung epithelial (A549) cells. Virol J (2011) 8:384. doi: 10.1186/1743-422X-8-384 21810281 PMC3162545

[22] Lanford RE Guerra B Lee H Chavez D Brasky KM Bigger CB. Genomic response to interferon-alpha in chimpanzees: Implications of rapid downregulation for hepatitis c kinetics. Hepatology (2006) 43(5):961–72. doi: 10.1002/hep.21167 16628626

[23] Irudayam JI Contreras D Spurka L Subramanian A Allen J Ren S. Characterization of type I interferon pathway during hepatic differentiation of human pluripotent stem cells and hepatitis c virus infection. Stem Cell Res (2015) 15(2):354–64. doi: 10.1016/j.scr.2015.08.003 PMC4600668 26313525

[24] Wu X Li S Chen D Zheng G Zhang Z Li Z. An inflammatory response-related gene signature associated with immune status and prognosis of acute myeloid leukemia. Am J Transl Res (2022) 14(7):4898–917. PMC9360836 35958446

[25] Alessandrini F Pezze L Ciribilli Y. Lamps: Shedding light on cancer biology. Semin Oncol (2017) 44(4):239–53. doi: 10.1053/j.seminoncol.2017.10.013 29526252

[26] Nagelkerke A Mujcic H Bussink J Wouters BG van Laarhoven HW Sweep FC. Hypoxic regulation and prognostic value of Lamp3 expression in breast cancer. Cancer (2011) 117(16):3670–81. doi: 10.1002/cncr.25938 21319150

[27] Elliott B Scolyer RA Suciu S Lebecque S Rimoldi D Gugerli O. Long-term protective effect of mature dc-lamp+ dendritic cell accumulation in sentinel lymph nodes containing micrometastatic melanoma. Clin Cancer Res (2007) 13(13):3825–30. doi: 10.1158/1078-0432.CCR-07-0358 17606713

[28] Zhang Z Ji W Huang J Zhang Y Zhou Y Zhang J. Characterization of the tumour microenvironment phenotypes in malignant tissues and pleural effusion from advanced osteoblastic osteosarcoma patients. Clin Transl Med (2022) 12(11):e1072. doi: 10.1002/ctm2.1072 36305631 PMC9615475

[29] Oh SA Wu DC Cheung J Navarro A Xiong H Cubas R. Pd-L1 expression by dendritic cells is a key regulator of T-cell immunity in cancer. Nat Cancer (2020) 1(7):681–91. doi: 10.1038/s43018-020-0075-x 35122038

[30] Zhang Q He Y Luo N Patel SJ Han Y Gao R. Landscape and dynamics of single immune cells in hepatocellular carcinoma. Cell (2019) 179(4):829–45.e20. doi: 10.1016/j.cell.2019.10.003 31675496

[31] Sun K Xu R Ma F Yang N Li Y Sun X. Scrna-seq of gastric tumor shows complex intercellular interaction with an alternative T cell exhaustion trajectory. Nat Commun (2022) 13(1):4943. doi: 10.1038/s41467-022-32627-z 35999201 PMC9399107

[32] Liu T Liu C Yan M Zhang L Zhang J Xiao M. Single cell profiling of primary and paired metastatic lymph node tumors in breast cancer patients. Nat Commun (2022) 13(1):6823. doi: 10.1038/s41467-022-34581-2 36357424 PMC9649678

[33] Cheng S Li Z Gao R Xing B Gao Y Yang Y. A pan-cancer single-cell transcriptional atlas of tumor infiltrating myeloid cells. Cell (2021) 184(3):792–809.e23. doi: 10.1016/j.cell.2021.01.010 33545035

[34] Du Y Cai Y Lv Y Zhang L Yang H Liu Q. Single-cell rna sequencing unveils the communications between malignant T and myeloid cells contributing to tumor growth and immunosuppression in cutaneous T-cell lymphoma. Cancer Lett (2022) 551:215972. doi: 10.1016/j.canlet.2022.215972 36265653

[35] Chen Z Zhou L Liu L Hou Y Xiong M Yang Y. Single-cell rna sequencing highlights the role of inflammatory cancer-associated fibroblasts in bladder urothelial carcinoma. Nat Commun (2020) 11(1):5077. doi: 10.1038/s41467-020-18916-5 33033240 PMC7545162

[36] Chang L Wang Z Li S Chen X Li X Zhao J. Type 2 inflammation suppression by T-regulatory cells attenuates the eosinophil recruitment in mucosa of chronic sinusitis. Clin Sci (Lond) (2020) 134(2):123–38. doi: 10.1042/CS20190388 31922185

[37] Abdellrazeq GS Fry LM Elnaggar MM Bannantine JP Schneider DA Chamberlin WM. Simultaneous cognate epitope recognition by bovine Cd4 and Cd8 T cells is essential for primary expansion of antigen-specific cytotoxic T-cells following ex vivo stimulation with a candidate mycobacterium avium subsp. paratuberculosis peptide vaccine. Vaccine (2020) 38(8):2016–25. doi: 10.1016/j.vaccine.2019.12.052 31902643

