# Supplementary material for: LAMP3 expression in the liver is involved in T cell activation and adaptive immune regulation in hepatitis B virus infection
Source: Front Immunol. 2023 Mar 16;14:1127572. doi: 10.3389/fimmu.2023.1127572 (PMC10060507; doi:10.3389/fimmu.2023.1127572)
Supplement: Supplementary file 1 [file DataSheet_1.docx]

Supplementary Material

LAMP3 expression in the liver is involved in T cell activation and adaptive immune regulation in hepatitis B virus infection

**Zilong Wang^1^†, Xiaoxiao Wang^1^†, Rui Jin^1^, Feng Liu^1^, Huiying Rao^1^, Lai Wei^2^, Hongsong Chen^1^, Bo Feng^1^***

*** Correspondence:** Bo Feng, [xyfyfb_1@sina.com](mailto:xyfyfb_1@sina.com)

# Supplementary Figures and Tables

For more information on Supplementary Material and for details on the different file types accepted, please see [here](https://www.frontiersin.org/guidelines/author-guidelines#supplementary-material).

## Supplementary Figures 1


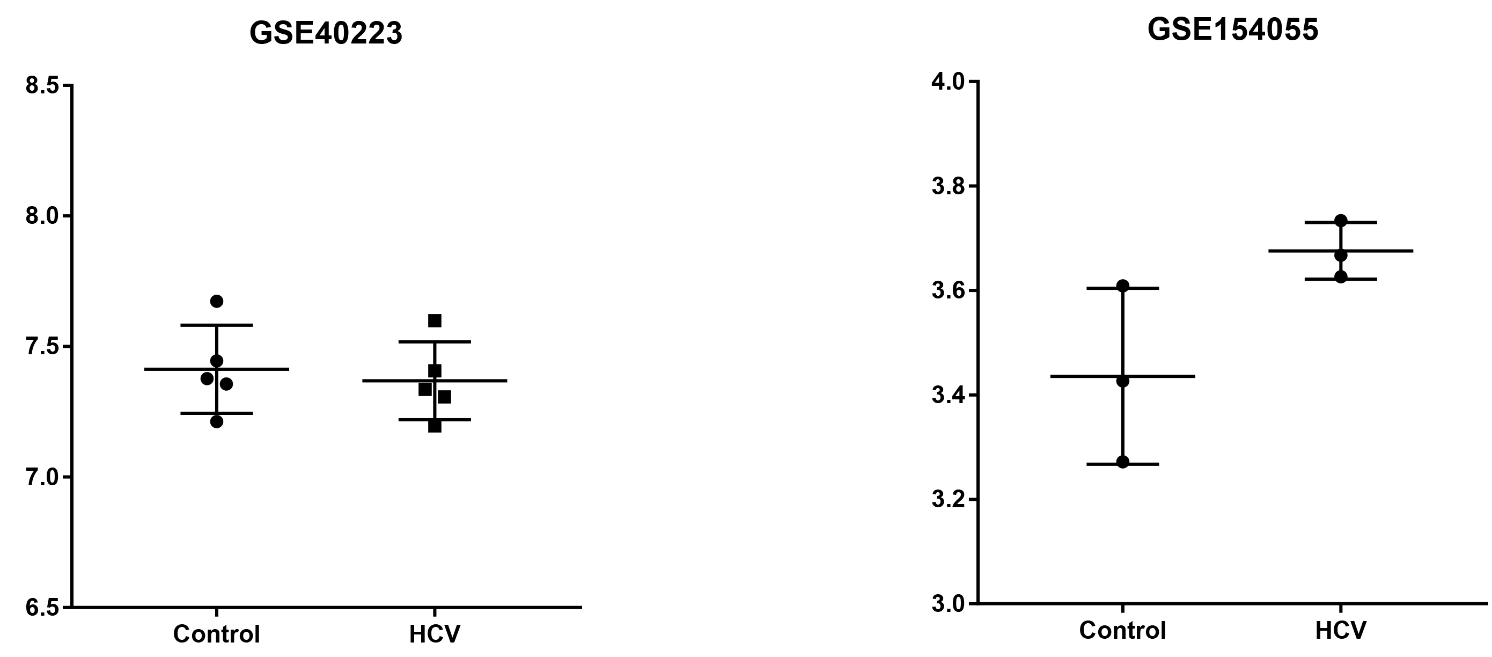


**Supplementary Figure 1.** The LAMP3 expression in HCV patients. The comparison of LAMP3 transcriptional level between control and HCV patients by Student’s t-test based on GEO database.

## Supplementary Figures 2

##
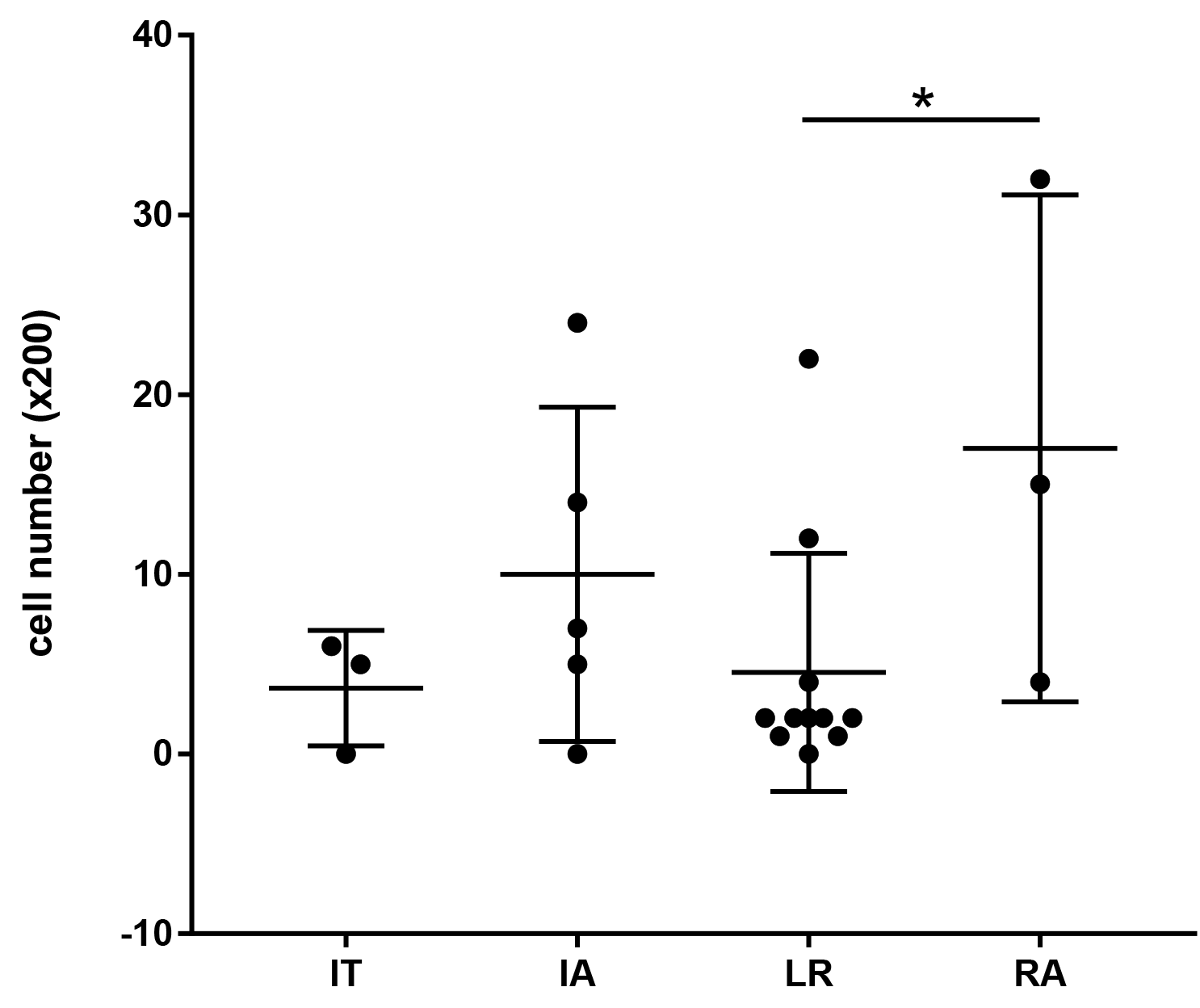


**Supplementary Figure 2.** The number of LAMP3+ cells in different natural history stages of chronic HBV infection. IT: Immune tolerant phase; IA: Immune activation; LR: Low replication; RA: Reactivation.
